# Patient-related post-ERCP pancreatitis (PEP) risk factors between two groups of patients: Prophylactic pancreatic stent and rectal indomethacin

**DOI:** 10.22088/cjim.13.4.728

**Published:** 2022

**Authors:** Hossein Ghalehnoei, Ahmad Hormati, Amir Houshang Mohammad Alizadeh, Sajjad Ahmadpour, Seyed Hassan Abedi

**Affiliations:** 1 Department of Medical Biotechnology, Molecular, and Cell Biology Research Center, School of Medicine, Mazandaran University of Medical Sciences, Sari, Iran; 2 Gastroenterology and Hepatology Diseases Research Center, Qom University of Medical Sciences, Qom, Iran; 3 Gastroenterology and Liver Diseases Research Center, Research Institute for Gastroenterology and Liver Diseases, Shahid Beheshti University of Medical Sciences, Tehran, Iran; 4 Cancer Research Center, Health Research Institute, Babol University of Medical Sciences, Babol Iran

**Keywords:** ERCP, Post ERCP pancreatitis, Pancreatic duct stent, Rectal indomethacin

## Abstract

**Background:**

Pancreatitis is one of the most crucial complications following endoscopic retrograde cholangiopancreatography (ERCP). The purpose of the current study was to investigate patient-related post-ERCP pancreatitis (PEP) risk factors in two groups of patients: prophylactic pancreatic stent and rectal indomethacin.

**Methods:**

Two different prophylactic modalities were planned and complications were assessed based on the defined inclusion criteria. In this study, the patients were evaluated for the procedure and patient-related risk factors in post-ERCP pancreatitis in the recipient groups of the prophylactic pancreatic stent and rectal indomethacin.

**Results:**

Pancreatitis was confirmed in 27 of all 170 selected patients after ERCP. By univariate analysis, two variables were significant with the development of PEP. Regarding the patient-related risk factors, unique subjects with common bile duct (CBD) dilated 10mm were more exposed to an increased chance of PEP (P=0. 015); meanwhile, other factors did not correlate with the increased possibility of PEP in both groups. The only procedure-related risk factor for PEP was the deep cannulation of the pancreatic duct in both groups during the procedure with an incremental significant incidence of pancreatitis (P=0.005). Comparison of prophylactic pancreatic stent and rectal indomethacin showed no effects in term of post ERCP pancreatitis reduction. Additionally, there was no significant difference between these two strategies in the rate of PEP.

**Conclusion:**

Prophylactic pancreatic duct stents and administration of rectal indomethacin cannot have particular approaches for reducing the possible occurrence of PEP. The increase in time of deep cannulation and the presence of CBD dilation <10mm could be considered as important risk factors.

Endoscopic retrograde cholangiopancreatography (ERCP) is one of the most effective procedures applied in the management of pancreaticobiliary disorders (1). The most important complications after this procedure include post- ERCP pancreatitis (PEP), infectious problems, hemorrhage, and perforation (2-4). One of the most common complications is acute pancreatitis that imposes critical problems like morbidity, occasional death, and increase of health care expenditures for the patients (5, 6). View to literatures, we find different incidence rate of PEP, depending on patient selection, which has been reported between 1-15.7% (7-9). Several risk factors including female sex, adolescence age, history of prior PEP, and the absence of chronic pancreatitis can increase the occurrence of PEP (10). 

So far, scientists put in to effort to find best solutions for the prevention of PEP; however, no ideal procedure or medication has been definitively verified for reducing post-ERCP pancreatitis. In this way, various preventive protocols including wire-guided biliary cannulation, placement of pancreatic duct stent and pharmacotherapy have been approved. Conflicting results about the usefulness of each mentioned methods have been reported in the literatures. It was reported that the placing of stent in the pancreatic duct could reduce the severity of PEP in high-risk patients (11, 12). On the other hand, any failure in the placement of the stent itself, can cause pancreatitis, perforation with bleeding and pain in the patients (13). As mentioned above, pharmacotherapy is another method for PEP prevention, as due to the role of inflammatory response in pathophysiological effects of PEP, inhibition of prostaglandins, phospholipase A2, and neutrophil endothelial interactions by NSAIDs can prevent PEP occurrence (14). Some study findings demonstrated that NSAIDs can reduce ERCP complications in patients (15-17). Rectal indomethacin was proposed as the other way to prevent PEP in high-risk patients (18). In this study, we aimed to compare two methods of prophylactic stent and rectal indomethacin used for the prevention of PEP in patients.

## Methods

This study was approved by the Ethics Committee of Babol University of Medical Sciences (ethical code: IR.MUBABOL.REC.1399.371). Writing informed consent was obtained from all patients. Out of 620 patients who referred to Taleghani Hospital in one year, between Jan 2019 and Jan 2020, 170 were candidates to undergo ERCP. The exclusion criteria were as follows; previous history of pancreatitis, interventional activities on the pancreatic duct, chronic pancreatitis, and history of ERCP failure. The reasons of the referred patients who underwent ERCP were as follows; pancreatic cancer (n=5), cholangiocarcinoma (n=29), ampullary cancer (n=6), benign strictures of the bile ducts (n=40) and gallstone (n=90). The provisional diagnosis of pancreatitis was applied, biliary stenting was performed in 47 subjects and a pancreatic stent was used in 53 cases.

In the present study, unintentional cannulation was considered as accidental as entering of sphincterotomy or guidewire inside the pancreatic duct. Complete tests, including ALT, AST, bilirubin, CRP, CBC and platelet counts were performed for the patients before the procedure. Once again, the serum amylase and lipase level were measured 24 h after post ERCP. Post ERCP pancreatitis (PEP) was defined as persistent abdominal pain following pancreatitis along with the increasing of serum amylase or lipase up to fold ≥3, after 24 hours post ERCP. In this study, all patients were divided into two subgroups; as, for one group we used the prophylactic PD stent and for the other, we used rectal indomethacin, and then we compared these procedures. 

**Statically analysis**: The chi-square test and/or Fisher’s exact test were used for univariate analysis of category data, also the student’s t-test was used for the analysis of quantitative data. The level of statistical significance was considered at p < 0.05.

## Results

**Fig 1:**
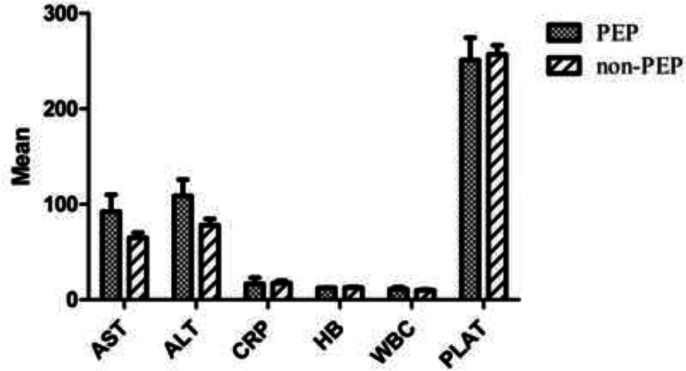
Comparison of pre ERCP laboratory finding variables between patients who had PEP complications and who did not. There were no significant differences found between the serum laboratory findings including AST, ALT, CRP, HB, WBC and PLT among patients

Abbreviations: AST: Aspartate transaminase; ALT: Alanine transaminase; CRP: C-reactive protein; HB: Hemoglobin; WBC: White blood cells; PLT: Platelet

170 patients who were candidates for ERCP (92/83: males/females) were included in the study. Demographic findings, medication history, and risk factors among the patients and their correlation with PEP incidence are given in table 1. No statically significant association was found between these variables and PEP increased. Comparison of the pre-ERCP laboratory finding variables between two subgroups: A) PEP (n=27) and B) without PEP (n=148) are given in table 2 and fig 1. The results did not show any significant difference between two groups. The relationship between the type of intervention procedure and the incidence of PEP is summarized in table 3. Overall, guidewire biliary cannulation was achieved successfully in 89.1 % (n=156) of patients. Additionally, biliary cannulation was performed incompletely (partial) in 7.4 % (n=13) and thoroughly failed in 3.4% (n=6). Deep cannulation was achieved in 10 minutes in 33.9% (n=53), between 10-30 minutes in 31.4 % (n=49) and in >30 minute in 34.6% (n=54) of patients. It is of interest that there was a direct and significant relationship between the increase of the time of deep cannulation with the incidence of PEP (table 3) (P=0.005). The performance of cleaning biliary stone in most of the patients was successful and only failed in 17.7% (n=31) of cases. Evaluation of serum lipase and amylase showed that serum (>100U/ml) and lipase amylase (>60U/ml) values were elevated in 70 (40%) and 73 cases (41.7%), 24h post ERCP. Three-fold serum amylase elevation was seen in 40 (22%) patients, and an increase in the value of lipase was found in 38 (21%) cases. The evaluation of post ERCP clinical symptoms indicated that 43 (24%) cases persistently suffered from abdominal pain. Threefold increase in one of the enzymes along with abdominal pain was seen in 27 patients.

**Table 1:** Comparison of the patient-related risk factors between two group patients: A) Prophylactic pancreatic (PD) stent and rectal indomethacin (B). The number of patients in each group were as follows: biliary stent (n=53), PD stent (n=47), rectal indomethacin (n=128), and incidence of PEP (n=27)

**variables**	**Biliary stent**	**PD stent (A)**	**Rectal Indomethacin (B)**	**Patients(n)**	**Incidence of PEP**	**P- value**
Female/male	26/27	26/21	57/71	83/92	11/16	0.44
Smoker (yes/no)	7/46	9/38	15/113	24/151	1/26	0.449
Age (<60years/>60years)	29/24	28/19	68/60	96/79	13/14	0.1
Alcoholism (yes/no)	1/52	1/47	2/126	3/172	1/26	0.38
Opium addict(yes/no)	2/51	4/43	5/123	9/166	1/26	0.7
Abdominal pain (yes/no)	39/14	33/14	69/23	118/57	19/8	0.723
Cholecystectomy (yes/no)	15/38	15/32	29/99	44/131	4/23	0.179
History of PEP (yes/no)	3/50	4/43	3/125	7/168	1/26	0.932
DM(yes/no)	7/46	5/42	17/111	22/153	2/25	0.379
Chronic-pancreatitis (yes/no)	3/50	1/46	6/122	7/168	2/25	0.326
Cardiopulmonary (yes/no)	12/41	8/39	21/107	29/146	4/23	0.79

*significant association

**Table 2:** Comparison of the pre-ERCP laboratory finding variables between two subgroups: A) PEP (n=27) and B) Without PEP (n=148). Data were reported as mean ± S.E.M

**Variables**	**PEP**	**Without PEP**	**P-value**
ALT	108.92**±**17	78**±**6.4	0.195
AST	92.3**±**17.7	64.9**±**5.1	0.087
AST /ALT	1.15**±**0.12	1.3**±**0.21	0.64
HB	12.3**±**0.35	12.6**±**0.75	0.86
WBC	11.05**±**2.17	9.9**±**0.71	0.56
CRP	16.7**±**6.5	17.4**±**2.5	0.91
PLAT	251.08**±**23.5	257.06**±**9.4	0.83

*significant association

**Table 3:** The relationship between the type of intervention procedure and the incidence of PEP

**Variables**	**Incidence of PEP**	**P-value**	**OR**	**95%CI**
Placement of stent				
Biliary stent	8(29.6%)	0.93	0.964	0.39-2.3
PD stent	6(22.2%)	0.55	0.74	0.281-1.9
Administration of rectal Indomethacin	21(77.8%)	0.55	1.3	0.5-3.5
PD Guidewire	12(44.4%)	0 .2	1.7	0.74-3.9
Time of deep cannulation		0.005*	-	-
<10min	6(22.2%)			
10-30 min	8(29.6%)			
>30 min	13(48.1%)			
Balloon dilation of S.O	7(25.9%)	0.6	1.2	0.47-3.1
Biliary sphincterotomy	16(59.3%)	0.82	0.9	0.39-2.1

Placing a PD stent (n=47), biliary stent (n= 53), administration of rectal indomethacin (n=128), biliary sphincterotomy (n=107), balloon dilation of S.O (n=40) and PD guidewire (n=59).

Table 4 shows the comparison of the ERCP frequency and relationships with an incidence of PEP in two groups underwent PD stent and rectal indomethacin. Findings did not establish any association between most of the ERCP finding variables with an incidence of PEP. It was confirmed that individuals with small common bile duct (CBD) diameter or ≤10mm obtained a higher risk for developing PEP than the patients who had the larger CBD diameter (≥10mm) (PEP: 20 *vs.* 7). Therefore, the diameter of small bile duct had a significant association with increases in the incidence of PEP during ERCP (P=0.015). Statistical analysis of intervention procedures did not demonstrate a significant correlation between the variables and the incidence of PEP. There was no significant association in rates of PEP between two groups who received rectal indomethacin and PD stent. Notwithstanding, 21 out of 27 cases with PEP complication was among the users of rectal indomethacin.

**Table 4:** The frequency of ERCP in subgroups and correlations of these findings with incidence of PEP. The number of patients in each group is as follows: biliary stent (n=53), PD stent (n=47), indomethacin (n=128), and incidence of PEP (n=27)

**Variables**	**Biliary stent**	**PD stent**	**indomethacin**	**Total of patients**	**Incidence of PEP**	**P- value**
GB dilation	1(1.9%)	1(2.1%)	4(3.1%)	5(2.9%)	1(3.7%)	0.77
CBD dilation	33(62.3%)	31(66%)	76(59.4%)	107(61.1%)	13(48.1%)	0.132
Diameter of CB Dilation						0.015*
<10mm	27(50.9%)	14(29.7%)	66(51.5%)	80(45.7%)	20(74%)	
>10mm	25(47.2%)	29(61.7%)	66(51.5%)	95(54.2%)	7(25.9%)	
CBD stricture	13(24.5%)	10(21.3%)	37(28.9%)	47(26.8%)	11(40.7%)	0.07
CBD stone	23(43.4%)	22(46.8%)	58(45.3%)	80(45.7%)	4(14.8%)	
PD dilation	3(5.7%)	5(10.6%)	5(3.9%)	10(5.7%)	1(3.7%)	0.62
PD irregularity	2(3.8%)	3(6.4%)	3(2.3%)	6(3.4%)	1(3.7%)	0. 93
Malignancy	9(17%)	10(21.2%)	30(23.4%)	40(22.9%)	8(29.6%)	0.36
Pancreas	2(3.7%)	4(8.5%)	1(0.7%)	5(2.8%)	1(12.5%)	
CCA	6(11.3%)	4(8.5%)	25(19.5%)	29(16.5%)	7(87.5%)	
Ampullary	1(1.8%)	2(4.2%)	4(3.1%)	6(3.4%)	0	
Diverticulum	2(3.8%)	0	8(6.3%)	8(4.6%)	0	0.81
CBD cyst	1(1.9%)	1(2.1%)	1(0.8%)	2(1.1%)	0	0.55

*significant association

## Discussion

In this study, the role of short-term prophylactic pancreatic stents and rectal indomethacin to prevent ERCP-induced pancreatitis (PEP) was compared in patients with distinct biliary disorders. It was found that several factors can increase the risk of PEP after ERCP. Extensive research in different countries was performed to find the risk factors to reduce ERCP side effects by utilizing various types of interventional procedures such as guide wires, consumption rectal indomethacin and placing PD stent. Cheon, Y et al’s. study showed that younger age (<65 years) and female patients were significantly associated with an increased risk of PEP. However, findings agree with the other studies (19, 20); as, our study did not confirm these findings. Similar to our findings, in Cheng et al.’s study, no significant association was reported between some risk factors including age and sex (21). Although the relationship between past medical history (e.g. PEP) and the absence of chronic pancreatitis with PEP were reported in some studies (10, 22), as our findings did not reveal this association. In several studies, increasing in the levels of inflammatory markers between 24-48h post-ERCP was used as a predictive factor (23, 24). In a few studies, the pre-ERCP level of inflammatory markers have been suggested as a potential risk factor for predicting PEP. Mohammad Alizadeh et al. found that the elevation of the pre-procedure ESR (>30 mm/h) can be considered as a significant factor for predicting the increased risk of PEP (2). In the present study, measurement of the pre-ERCP level of biochemical markers (ALT, AST, AST/ALT, CRP, WBC, and HB) did not show any significant difference between patients who present PEP and who did not. It seems that some procedures like biliary sphincterotomy and balloon dilation S.O could increase the risk of PEP (5, 20). Nevertheless, our findings did not confirm this association. 

Several studies have reported that wire-guided biliary cannulation is associated with the reduced incidence of PEP (25-27). However, in agreement with the current study, this association was not found in Mariani A. et al.’s study (28). Notwithstanding, they did not find a correlation between the use of wire -guided biliary cannulation by increasing the risk of pancreatitis, but our findings clearly showed a direct and significant association between the time of deep cannulation and the increased risk of pancreatitis. It seems that excess time spend for procedure performance, and the difficult cannulation on the other hand, can prompt inflammation. These findings are in agreement with Freeman L. et al.’s findings that shows the relationship between difficult cannulation and pancreatitis (20). In several studies, it was proposed that the placing of prophylactic PD stent can reduce the risk and symptoms of pancreatitis in patients which was mediated by the increase of free flow of pancreatic exocrine secretions and consequently decreasing ductal hypertension; however, these findings were not verified in our study. Recently, in some studies, the role of anti-inflammatory drugs has been reported for reducing the risk and symptoms of PEP (8, 14, 16), although the other studies did not approve the usefulness of these agents (29, 30). Absence of any significant correlation between using rectal indomethacin and reducing PEP was the other finding of our study. In current study, we showed that patients with CBD dilation <10mm were more exposed to PEP than the other groups (table 4). View to literatures, we could not find any relationship between small bile duct diameter (<5mm) and pancreatitis (20); but our study indicated that CBD dilation <10mm considerately was one important factor to increase the risk of PEP.

In this study, we find that there is no statistically significant correlation between demographic and past medication risk factors with the incidence PEP. Also, we did not find any relationship between applied interventional procedures and the increase or decrease of the risk of pancreatitis. There was no significant difference in the PEP among individuals who received both prophylactic PD stent and rectal indomethacin. It seems that small bile duct could increase the risk of pancreatitis, so it is suggested that the procedure should be avoided during second ERCP. Also, prophylactic PD stenting was proposed for the first CBD cannulation, but it may be possible that the guidewire accidentally entered inside the PD, the occurrence of PEP in the patients.
